# Dietary niacin supplementation improves meat quality, muscle fiber type, and mitochondrial function in heat-stressed Taihe black-bone silky fowls

**DOI:** 10.3389/fvets.2024.1491553

**Published:** 2024-10-14

**Authors:** Wenliang Mei, Wenyan Zhang, Ziyu Hu, Mingren Qu, Gen Wan, Xiaoquan Guo, Chuanbin Chen, Lanjiao Xu

**Affiliations:** Jiangxi Key Laboratory of Animal Nutrition, Nanchang, Jiangxi, China

**Keywords:** Taihe black-boned silky fowl, niacin, heat stress, meat quality, mitochondrial biogenesis

## Abstract

**Background:**

A recent study has shown that niacin supplementation induces the conversion of type II to type I muscle fibres, thereby promoting a phenotypic shift in oxidative metabolism in porcine skeletal muscle. These effects may be mediated by modulation of the AMPK1/SIRT1 pathway, which activates peroxisome proliferator-activated receptor *γ* coactivator-1α (PGC-1α), a key regulator of fibre conversion, thereby promoting skeletal muscle mitochondrial biogenesis and myofibre conversion. In this study, we explored how niacin (NA) supplementation impacts the quality of meat and the characteristics of muscle fibers in Taihe Black-bone Silky Fowls (TBsf) exposed to heat conditions.

**Methods:**

Chickens were rationally assigned to five different treatment groups with five replicates of six chickens each: thermophilic (TN), heat stress (HS) and HS + NA (HN) groups, with the HN group being supplemented with 200, 400 and 800 mg/kg (HS + NA_0.02_, HS + NA_0.04_ and HS + NA_0.08_) NA in the premix, respectively.

**Results:**

The results of the experiment showed that addition of 800 mg/kg NA to the diet significantly improved TBsf muscle tenderness compared to HS. Dietary enrichment with 200-800 mg/kg NA significantly increased total antioxidant capacity, superoxide dismutase, and glutathione peroxidase activities, while significantly decreasing malondialdehyde compared to HS. Incorporation of 200-800 mg/kg NA into the diet significantly reduced lactate dehydrogenase activity and myosin heavy chain (MyHC-IIB) gene expression. Furthermore, adding 800 mg/kg NA can significantly enhance the mRNA expression of mitochondrial transcription factors (TFAM and TFB1M) in TBsf skeletal muscle. Adding 400 and 800 mg/kg of NA significantly increased the mRNA expression of AMP-activated protein kinase 1 (AMPK1), PGC-1α, cytochrome c oxidase (Cytc), and nuclear respiratory factor (NRF-1) in the skeletal muscle of TBsf. Supplementing NA at 200-400 mg/kg significantly increased the expression of Sirtuin 1 (SIRT1) mRNA in TBsf skeletal muscle.

**Conclusion:**

The experimental results showed that the addition of NA to the diet reduced the shear force of TBsf muscle under heat exposure conditions. It increased the proportion of type I muscle fibres by increasing the antioxidant capacity of the muscle and by promoting mitochondr fibreial biogenesis. Considering the results of this study, it is recommended that TBsf be supplemented with 400-800 mg/kg of NA in the diet to reduce the adverse effects of heat stress on meat quality.

## 1 Introduction

Poultry is the second most consumed meat globally. However, the intense focus on accelerating the growth rate of poultry and the various stresses involved in chicken meat production have led to a decline in the quality of poultry meat. Poultry meat quality is susceptible to a variety of stressors, with high temperature being one of the most significant threats. HS increases the production of mitochondrial reactive oxygen species in chicken skeletal muscle, leading to oxidative stress, which in turn can cause lipid peroxidation and protein damage, shortening the shelf-life of the meat and adversely affecting the quality of poultry meat (1). Therefore, improving meat quality through nutritional adjustments has become an important topic in the realm of animal research.

The main evaluation indicators for meat include sensory characteristics (meat color, tenderness and taste) as well as physical and chemical characteristics. Meat color is the most intuitive indicator of product freshness and an important commercial characteristic that influences consumer purchasing decisions. Tenderness is a key factor influencing consumer satisfaction (2). pH is one of the most important indicators of meat quality, and likewise one of the most important indicators of the degree of glycolysis in postmortem muscle. The composition of fiber types affects muscle characteristics. Different muscle fiber types exhibit differences in, color, drip loss, and shear, which are key factors in evaluating meat quality (3, 4). Myofiber types are determined by MyHC expression, including MyHC1, MyHC2, and MyHC2B (5, 6). Oxidized muscle fibers are enriched in myoglobin, mitochondria and cytochromes, resulting in a more attractive appearance (7). Similarly, oxidized muscle fibers have a higher intramuscular fat content (8) and greater muscle tenderness (9). Many mitochondria are present in the muscle and are mainly involved in the contraction and metabolism of muscle fibers. Therefore, the conversion of muscle fibers is inevitably accompanied by mitochondrial biogenesis (10, 11). The make-up of muscle fibers is dynamic and highly plastic, but type conversion of muscle fibers must follow a path: I → IIA → IIB (12). It is well established that mitochondria play a crucial role in regulating the size, metabolism and function in skeletal muscle fibers (13). It was found that mitochondria may be key regulators of skeletal muscle fiber types (14). Previous studies have emphasized the expression of genes within mitochondria, revealing key roles for AMPK, SIRT1, and PGC-1α in regulating energy conversion (15) and regulates the distribution of muscle fibers in the muscle (16). SIRT1 acts to promote mitochondrial biosynthesis in muscle (17). In addition, SIRT1 activates PGC-1α by increasing mitochondrial function and number through deacetylation (18, 19), a key regulator associated with muscle fiber (20). AMPK has been demonstrated to enhance mitochondrial function and oxidative capacity while preventing mitochondrial dysfunction (21).It has been shown that the addition of 1.0% arginine to the diet significantly increases the expression of Cytc and PGC-1α mRNA, enhances the function and number of mitochondria in skeletal muscle, and thus increases the percentage of type I muscle fibers (5). In conclusion, the role of mitochondrial biogenesis is crucial in promoting myofiber transformation. Broilers are particularly susceptible to the influence of heat stress during the growth phase due to their feather-covered body and absence of sweat glands. HS has a detrimental effect on meat quality. Therefore, we need to address the question of how to find effective means to alleviate the deterioration in poultry meat quality caused by heat stress. Many studies have shown that supplementation with taurine (22) as well as plant extracts such as Pueraria mirifica (23) and resveratrol (24) can improve meat quality.

NA, the vitamin with the simplest structure, has multiple functions. These functions include anti-inflammatory properties, antioxidant effects, and a regulatory role in lipid metabolism (25–27). NA has been reported to reduce heat stress by upregulating heat shock protein-70 mRNA expression (28), NA was added to the diet to promote vasodilation and reduce skin temperature in Holstein cows during heat stress (29). NA has important roles in the regulation of energy metabolism, methylation, DNA repair and immune function (30), which may have positive effects in the prevention and control of HS. The addition of NA to diets has been reported to reduce carcass shrinkage and muscle yellowness values, increase water holding capacity, mitigate the pH_24h_ decline, and improve meat quality (31). However, the reasons why NA improves meat quality in HS and whether this improvement is related to the regulation of muscle myofiber type by NA remain unclear. Therefore, the aim of this experiment was to investigate the effects of NA on meat quality and muscle fiber type in HS broilers.

## 2 Materials and methods

### 2.1 Ethics statement

Animal husbandry and experiments were conducted in accordance with the Chinese Animal Welfare Guidelines approved by the Jiangxi Agricultural University Animal Care and Use Committee (loan number JXAULL-2021 1,213).

### 2.2 Experimental design

In the present study, 150 healthy male TBsf with a mean weight of 2802.00 ± 226.8 g were selected. These chickens were rationally assigned to five different treatment groups with five replicates of six chickens each: thermophilic group (TN), heat stress group (HS) and HS + NA group (HN) with 200, 400 and 800 mg/kg NA added to the premixes, respectively. The temperature of the TN group was maintained at 24 ± 2°C, whereas the temperature of the HS and HN group increased to 34 ± 2°C between 09:00 and 17:00 h. The temperature was maintained at 24 ± 2°C for the rest of the day, and feeding was started at 7:00 h. Nutritional levels in the diets were in accordance with the guidelines established by the National Research Council (1994), and the specific composition and nutrient content of the basal diets are shown in Table 1. Temperature control was achieved using an electric heater and thermostat, and a humidifier ensured that the relative humidity was maintained at between 65 and 72%. The experiment was divided into a pre-test period (7 d) and a main test period (28 d) for a total of 35 days. The experiment was conducted at the Animal Nutrition Research Base of Jiangxi Agricultural University.

**Table 1:** Composition and nutrient levels of the basal diet (air-dry basis).

Items	
Ingredients	(%)
Corn	67.60
Soybean meal	22.00
Fish meal	2.00
Soybean oil	4.00
Dicalcium phosphate	1.00
Limestone	1.10
_DL_-Methionine	0.09
Threonine	0.05
Salt	0.30
Zeolite powder	1.36
Premix^*^	0.50
Total	100.00
Nutrient levels (%)	
Metabolizable energy (MJ/kg)	12.97
Crude protein	16.19
Calcium	0.81
Available phosphorus	0.38
Threonine	0.69
Lysine	0.86
Methionine	0.36
Methionine + cystine	0.65

Provided per kilogram of diet: vitamin A 11250 IU, vitamin D3 3,750 IU, vitamin E 30 IU, vitamin K 1.75 mg, thiamin 2.75 mg, riboflavin 9.30 mg, calcium pantothenate 18.75, niacin 37.50, vitamin B6 3.75 mg, biotin 0.15 mg, folic acid 1.80 mg, vitamin B12 0.028 mg, choline chloride 720 mg, Fe (from ferrous sulfate) 80 mg, Cu (from cupric chloride) 8 mg, Mn (from manganous sulfate) 80 mg, Zn (from Zinc sulfate) 60 mg, I (from calcium iodate) 0.35 mg, Se (from sodium selenite) 0.15 mg.

### 2.3 Serum biochemical indicators

At the end of the test, a TBsf of near average weight (fasted for 12 h) was selected. Blood was collected from the blood tube, which was allowed to stand for 30 min at an angle of 45°. The blood was then centrifuged for 10 min.

### 2.4 Antioxidant status assay

Precision weighing of the leg muscle tissue is conducted, followed by the dilution with physiological saline at a 1:9 ratio of tissue mass to saline volume, using saline at 4°C. Subsequently, the tissue is homogenized at-20°C using a high-speed homogenizer. Quantification of total protein (TP) content in leg homogenates. Concurrently, the activities of SOD, GSH-Px, and the T-AOC are assessed, alongside the determination of MDA content. The requisite assay kits for these measurements were procured from the Nanjing Jian Cheng Bioengineering Institute.

### 2.5 Meat quality

The leg muscles were removed within 45 min after slaughter. A cut was made with a scalpel at different locations on the surface of the leg muscles to determine the PH, shear force, water holding capacity and tenderness of the muscles (23). The pH of three different sites was assessed using a pH meter (HI9125 Hanna, Italy) and the average of three replicates was calculated by inserting the electrode tip into the meat. After 24 h of refrigeration at 4°C, the pH of the leg muscles was reassessed using the same method. Meat color was determined using a meat colorimeter (CR410, Konica Minolta, Japan), ensuring that bruising and fat were excluded from the measurement. Three recordings were made and averaged. Leg muscle samples were trimmed into 2 × 3 × 4 cm pieces, with the surface fascia and fat removed, and weighed to record the initial weight. The samples were placed in self-sealing bags and stored in a refrigerator at 4°C, ensuring that there was no contact between the sample and the bag. A total of 24 h later, the samples were re-weighed and this measurement was recorded as the final mass. To assess cooking losses, place the sample in a bag, immerse it in a thermostatic water bath and place a thermometer as required. When the thermometer indicated 70°C, cooled and weighed again with paper to absorb the water. Then, several small strips were cut with a scalpel in the direction of the muscle fibers. These small strips were then sliced perpendicular to the muscle fibers using a tenderizer (9CL-ML3, Meiou, China) and the shear force was recorded.

### 2.6 Crude protein and intramuscular fat contents measurement

One right leg muscle was taken from each replicate and one leg muscle was taken for analysis. The moisture content of the hamstrings was determined using a freeze dryer; the crude fat (EE) content of the hamstrings was determined using Soxhlet extraction; while the crude protein (CP) content of the hamstrings was determined using Kjeldahl nitrogen determination (32).

### 2.7 Metabolic enzyme activities assay

Weigh exactly 0.5 g of chicken leg muscle and add cold saline solution at 4°C in the ratio of 1 g of muscle tissue to 9 milliliters of saline. Homogenize the tissue at −20°C using a homogenizer to produce a chicken leg muscle homogenate. Follow the kit instructions carefully to quantify total protein (TP) in the homogenate. Additionally, determine the activities of succinate dehydrogenase (SDH), malate dehydrogenase (MDH), and lactate dehydrogenase (LDH) in the supernatant according to the kit’s protocol. The test kits were purchased from Nanjing Jian Cheng Bioengineering Institute.

### 2.8 qPCR

#### 2.8.1 The isolation of RNA and the subsequent generation of complementary DNA (cDNA)

Accurately weigh 100 mg of the leg muscle tissue sample and combine it with 1 mL of TransZol reagent solution and three grinding beads. Homogenize the mixture at-20°C using a high-speed homogenizer to produce a leg muscle tissue homogenate. Next, follow the total RNA extraction protocol provided with the TransZol Up Plus RNA kit. Assess RNA integrity and quantity using a microvolume spectrophotometer (Nano Drop 2000 model) and document RNA concentration. Next, perform reverse transcription in strict accordance with the cDNA Synthesis SuperMix Kit from Quanshi Jin Biotechnology Co., Ltd., Beijing to synthesize cDNA for subsequent experimental applications (see Table 2 for details of the reverse transcription system).

**Table 2:** Reverse - transcription systems and conditions.

Reagent	Volume	Temperature	Time
Total RNA/mRNA	≤1 μg/≤100 ng	50°C	5 min
*5 × TransScript* Uni All-in-One			
SuperMix for qPCR	4 μL	85°C	5 s
gDNA remover	1 μL		
RNase-free water	Variable		

#### 2.8.2 qPCR assay

Adhere to the protocol provided with the Perfect Start™ Green qPCR SuperMix kit (Quan Shi Jin, Beijing) for the execution of the qPCR assay. The reaction mixture is composed of 2× Perfect Start™ Green qPCR SuperMix (10 μL), nuclease-free water (7.2 μL), cDNA template (2 μL), and equimolar concentrations of forward and reverse primers (0.4 μL each). The qPCR amplification protocol is initiated with a pre-denaturation step at 94°C for 30 s, followed by 42 cycles of denaturation at 94°C for 5 s, and annealing/extension at 60°C for 30 s. In this study, *β*-actin serves as the endogenous control gene, with the corresponding primer sequences detailed in Table 3.

**Table 3:** Primer sequences of genes.

Gene name	Accession number	Primer sequence (5′-3′)
*AMPK a 1*	NM_001039603.2	F:TGTCACAGGCACATGGTAGT R:TACTTCCGGTGCAGCATAGT
*SIRTI*	XM_046920057.1	F:GGAAATCTACCCAGGCCAGT R:GATCAGGCAGGAAGCTGTTG
*SIRT3*	XM_015286200.4	F:CAGACAAGGTCCCTCACTGT R:TCGCCAAAGAACACGATGTC
*PGC-la*	XM_046932881.1	F:AAAGACGTCCCTGCTCTGAA R:GCTGCTGTTCCTGTTCTCTG
*TFAM*	XM_046919713.1	F:ACGAGGAAGCAAGGAAGACA R:TTGAAGCCACTTCGAGGTCT
*TFBIM*	XM_046923024.1	F:AAGGAAGTTGCAGAGGTGGA R:GGCTGCTGTATCTTCGGTTG
*NRF1*	XM_046907008.1	F:ACACAGCAACAGACCACAAC R:AACCTGGATGAGGGACACAG
*Cytc*	NM_001398298.1	F:AAGCACAAGACTGGACCCAA R:AAGAGAAGCCCTCAGCTTGT
*COX5A*	XM_046925108.1	F:ACAGAAAGGATCGGCTTGGA R:GCGGGCATCAAACTCTTCAT
*ATP5a1*	AF332870.1	F:AGTTGGCTCAGTACCGTGAA R:GCACACCACGATTTAGCAGT
*MyHC I*	NM_001319304.2	F:TGAAGGAGCTCAGCTACCAG R:CACCTTCAGCTGCAGTTTGT
*MyHC II*	XM_046929275.1	F:AGCTGAACCAGATCAAGGCT R:CAGCTGGATCTCCATCTCGT
*MyHC II B*	XM_046906963.1	F:TGCTGACTGACCGTGAGAAT R:TIGATCCTCAAGCGTTCCCT
*β-actin*	NM_173979.3	F:CCCTGGAGAAGAGCTACGAG R:CAGGAAGGAAGGCTGGAAGA

### 2.9 Statistical analysis

Initially, all datasets underwent normality testing via the SAS procedure “proc univariate; data = test; normal.” Subsequent analyses included independent samples *t*-tests comparing the TN and HS treatments, as well as one-way ANOVA for the HS treatment groups, conducted using SPSS version 23.0. A *p* < 0.05 was deemed significant, while a *p* < 0.01 was considered highly significant.

## 3 Results

### 3.1 Serum biochemical indicators

**Figure 1:**
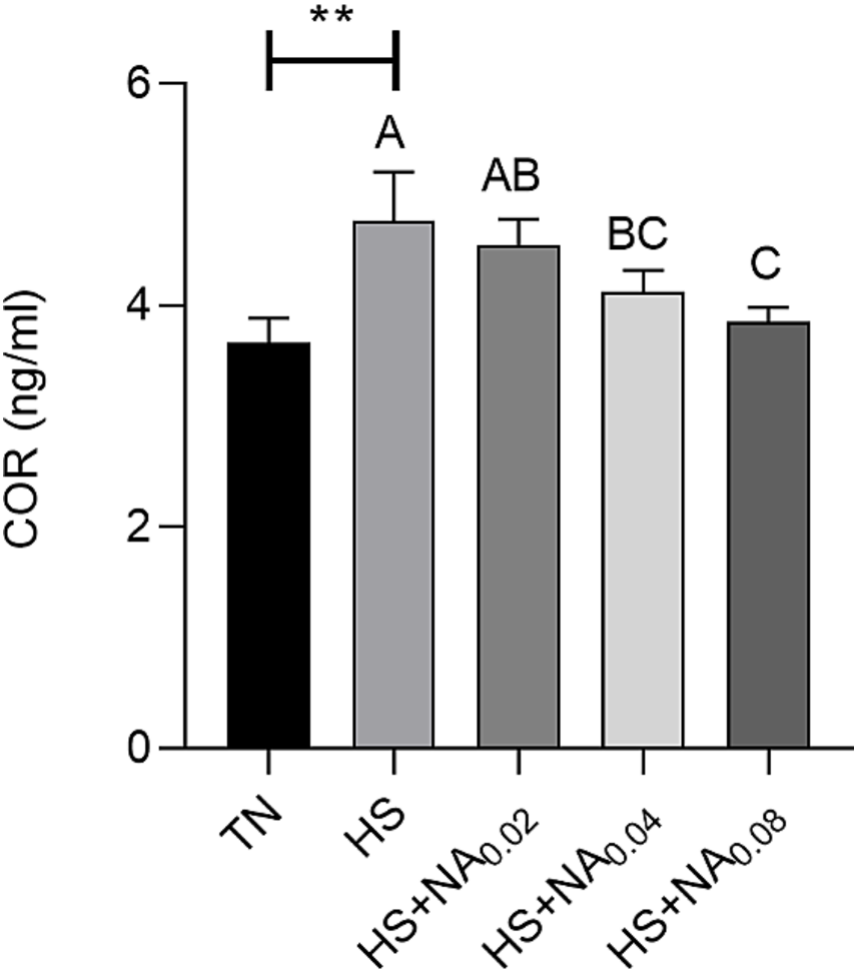
Effect of NA on COR in heat-stressed TBsf. Between HS and HS + NA groups, the difference between different lower letters reached a significant level (*p* < 0.05) and different upper letters reached an extremely significant level (*p* < 0.01). Not if data labeled with the same letter (*p* > 0.05). *p* <0.05 for the TN vs. HS group is indicated by *.

As can be seen from Figure 1, HS significantly increased the content of corticosterone (COR) of TBsf (*p* < 0.05). The inclusion of 400 and 800 mg/kg of NA in the diet significantly reduced serum COR levels compared to the HS group (*p* < 0.01).

### 3.2 Antioxidant index

Analysis of Table 4 indicates that, in comparison with the TN, heat stress significantly elevated the MDA levels (*p* < 0.001), concurrently leading to a pronounced reduction in the GSH-PX, T-AOC, and the SOD within the TBsf (*p* < 0.001). In contrast to the HS, dietary supplementation with 200–800 mg/kg of NA significantly mitigated the MDA levels in the leg muscle of the TBsf (*p* < 0.05), Addition of 400,800 mg/kg NA significantly increased T-AOC levels (*p* < 0.01). The dietary addition of 200, 800 mg/kg of NA significantly enhanced GSH-PX activity, while dietary supplementation with NA substantially elevated the SOD activity in the leg muscle tissues of the TBsf (*p* < 0.01).

**Table 4:** Effects of NA on antioxidant indexes of leg muscles of heat-stressed TBsf.

Items	TN	HS	HS + NA_0.02_	HS + NA_0.04_	HS + NA_0.08_	*P*_1_	SEM	*P*_2_
T-AOC (mmol/g)	2.02	1.28^A^	1.37^A^	1.70^B^	1.56^B^	<0.001	0.07	<0.001
GSH-PX (nmol/mgprot)	23.99	14.08^a^	15.81^b^	14.99^ab^	15.53^b^	<0.001	0.47	0.011
SOD (U/mgprot)	308.07	256.75^A^	269.84^B^	278.41^B^	280.19^B^	<0.001	5.25	0.001
MDA (nmol/mgprot)	2.92	4.35^a^	3.86^b^	3.83^b^	3.96^ab^	<0.001	0.17	0.032

Between HS and HS + NA groups, the difference between different lower letters reached a significant level (*P* < 0.05) and different upper letters reached an extremely significant level (*P* < 0.01). Not if data labeled with the same letter (*P* > 0.05).

### 3.3 Meat quality

Upon examination of Table 5, it is evident that, in contrast to the TN, HS significantly elevated the shear force and dripping loss in the leg muscles of the TBsf (*p* < 0.05). In addition, HS resulted in a significant decrease in pH 24 h postmortem and increased leg muscle loss by cooking (*p* < 0.05). The addition of 800 mg/kg of NA to the feed significantly (*p* < 0.01) reduced leg muscle shear.

**Table 5:** Effect of NA on meat quality of heat-stressed TBsf.

Items	TN	HS	HS + NA_0.02_	HS + NA_0.04_	HS + NA_0.08_	*P_1_*	SEM	*P_2_*
pH_45min_	6.41	6.33	6.44	6 0.46	6.28	0.508	0.11	0.380
pH_24h_	6.20	5.77	5.81	5.87	6.09	0.020	0.17	0.284
Lightness, L*	39.37	42.00	39.32	41.25	42.71	0.145	1.70	0.236
Redness, a*	0.30	0.25	1.17	0.97	−0.25	0.890	0.53	0.056
Yellowness, b*	5.16	4.56	4.61	6.08	5.69	0.430	0.84	0.210
Dripping loss (%)	1.10	1.32	1.36	1.26	1.28	0.049	0.94	0.876
Cooking loss (%)	0.11	0.10	0.11	0.10	0.09	0.012	0.01	0.171
Shear force (kg)	1.55	1.74^A^	1.72^A^	1.66^A^	1.43^B^	0.047	0.07	0.002

Between HS and HS + NA groups, the difference between different lower letters reached a significant level (*P* < 0.05) and different upper letters reached an extremely significant level (*P* < 0.01). Not if data labeled with the same letter (*P* > 0.05).

### 3.4 General muscle nutrients

An analysis of Table 6 reveals that, in comparison to the TN, heat stress significantly diminished the crude protein content and concurrently led to a significant enhancement in the moisture content of the leg muscles of the TBsf (*p* < 0.05). The added 800 mg/kg NA dramatically (*p* < 0.05) raised the CP content of leg muscles compared to HS.

**Table 6:** Effect of NA on the chemical composition of leg muscles of heat-stressed TBsf (%).

Items	TN	HS	HS + NA_0.02_	HS + NA_0.04_	HS + NA_0.08_	*P*_1_	SEM	*P*_2_
Moisture	67.80	69.78	67.15	67.16	64.80	0.045	1.70	0.069
Crude protein	30.48	28.31^a^	30.50^ab^	30.92^ab^	33.55^b^	0.041	1.64	0.044
Crude fat	1.25	1.35	1.91	1.52	1.31	0.662	0.42	0.491

Between HS and HS + NA groups, the difference between different lower letters reached a significant level (*p* < 0.05) and different upper letters reached an extremely significant level (*P* < 0.01). Not if data labeled with the same letter (*P* > 0.05).

### 3.5 Metabolic enzyme activities

**Figure 2:**
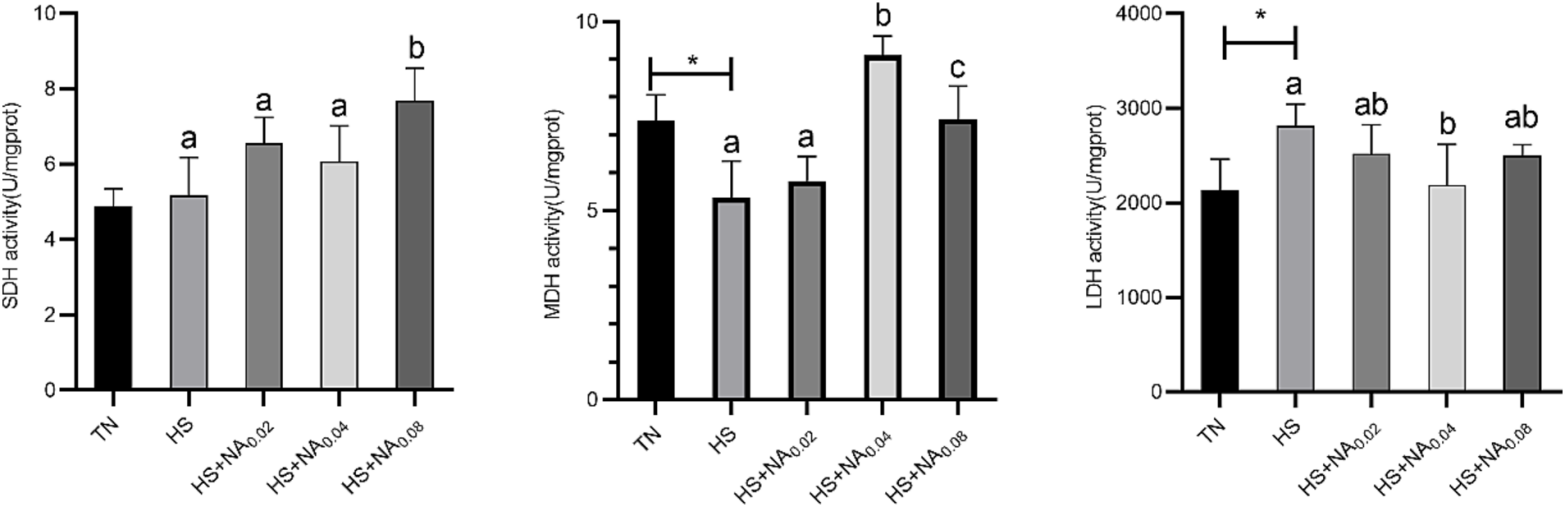
Effect of NA on the activity of muscle metabolizing enzymes in the leg muscles of heat-stressed TBsf. Between HS and HS + NA groups, the difference between different lower letters reached a significant level (*p* < 0.05) and different upper letters reached a very significant level (*p* < 0.01). Not if labeled with the same letter (*p* > 0.05). *p* < 0.05 for the TN vs. HS group is indicated by*.

Examination of Figure 2 elucidates that HS significantly elevated the activity of LDH in the leg muscle tissues while concurrently causing a significant reduction in the activity of MDH (*p* < 0.05). The added 800 mg/kg NA dramatically (*p* < 0.05) raised SDH activity. In addition, the added 400 and 800 mg/kg NA resulted in a dramatic increase in MDH activity (*p* < 0.05). Addition of 400 mg/kg NA markedly lowered LDH activity in TBsf leg muscle tissue (*p* < 0.05).

### 3.6 Muscle fiber type

**Figure 3:**
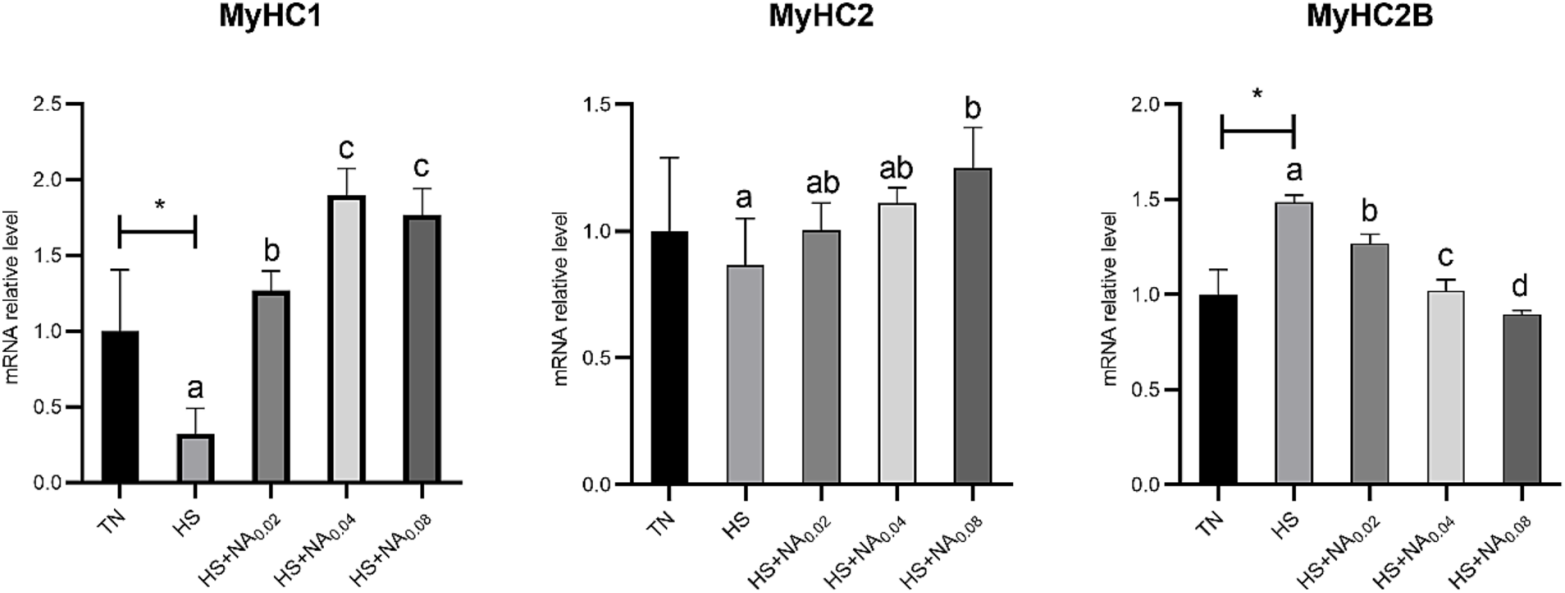
Effect of NA on the expression of MyHC gene in the leg muscles of heat-stressed TBsf. Between HS and HS + NA groups, the difference between different lower letters reached a significant level (*p* < 0.05) and different upper letters reached a very significant level (*p* < 0.01). Not if labeled with the same letter (*p* > 0.05). *p* < 0.05 for the TN vs. HS group is indicated by*.

Analysis of Figure 3 revealed that heat stress significantly down-regulated (*p* < 0.05) the level of MyHC1 mRNA and up-regulated (*p* < 0.05) the level of MyHC2B mRNA in the leg muscles of TBsf. In contrast to HS, the addition of 200–800 mg/kg NA markedly diminished the levels of MyHC2B mRNA in muscle, while significantly increasing the levels of MyHC1 mRNA (*p* < 0.05). In contrast to HS, supplementation with 800 mg/kg NA significantly increased MyHC2 mRNA content in TBsf leg muscles (*p* < 0.05).

### 3.7 AMPK1/SIRT1 pathway

**Figure 4:**
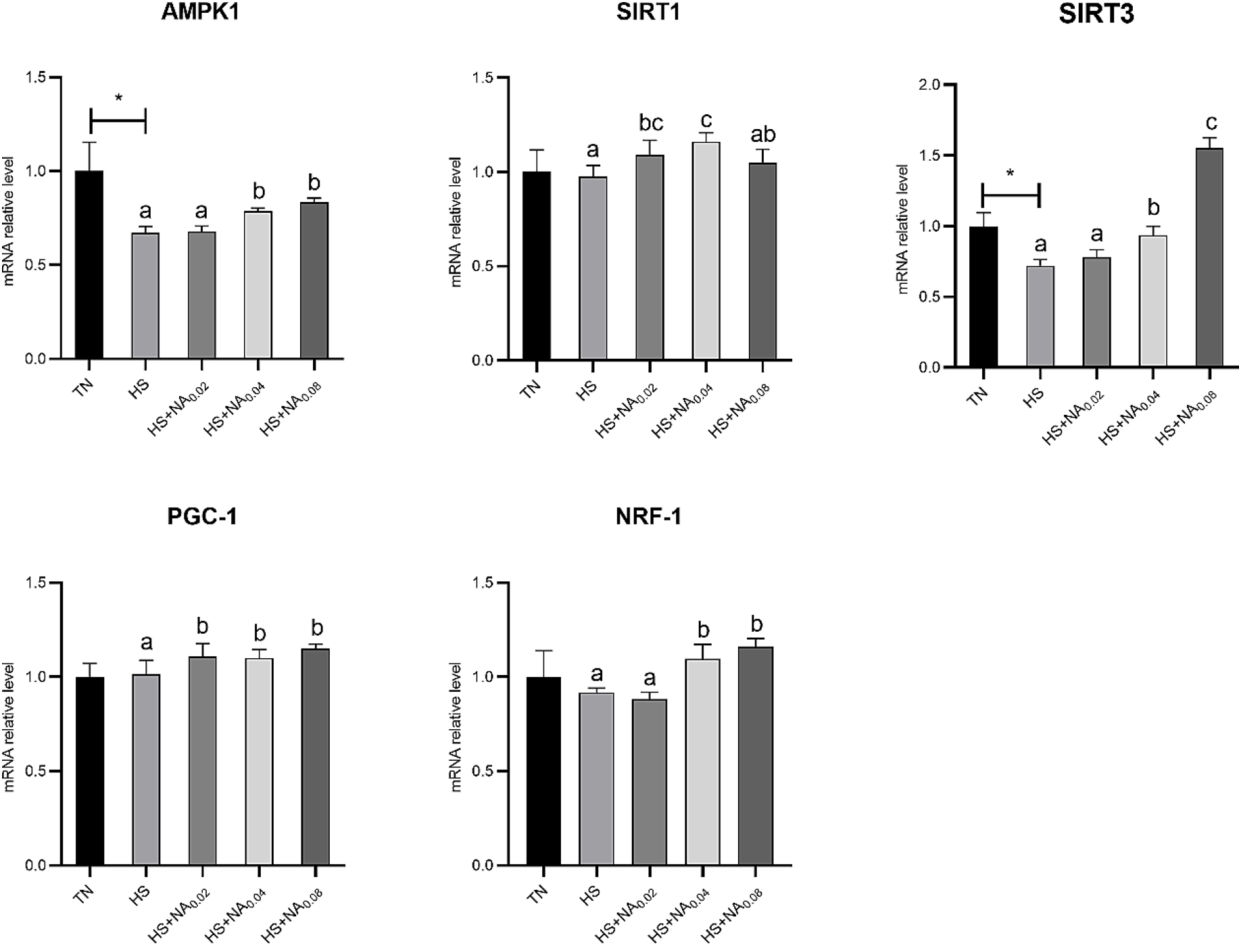
Effect of NA on the AMPK1/SIRT1 pathway in heat-stressed TBsf. Between HS and HS + NA groups, the difference between different lower letters reached a significant level (*p* < 0.05) and different upper letters reached a very significant level (*p* < 0.01). Not if labeled with the same letter (*p* > 0.05). *p* < 0.05 for the TN vs. HS group is indicated by*.

Figure 4 shows that HS significantly down-regulated the abundance of mRNAs for AMPKα1 and SIRT3 in TBsf skeletal muscle (*p* < 0.05). Adding NA markedly raised the mRNA expression levels of PGC-1α in TBsf muscle (*p* < 0.05). Abundance of mRNAs for AMPK1, SIRT3 and NRF-1 was dramatically higher (*p* < 0.05) with the addition of 400 mg and 800 mg/kg NA. Addition of 200 mg and 400 mg/kg NA also dramatically enhanced the abundance of SIRT1 mRNA in skeletal muscle (*p* < 0.05).

### 3.8 Mitochondrial biogenesis

**Figure 5:**
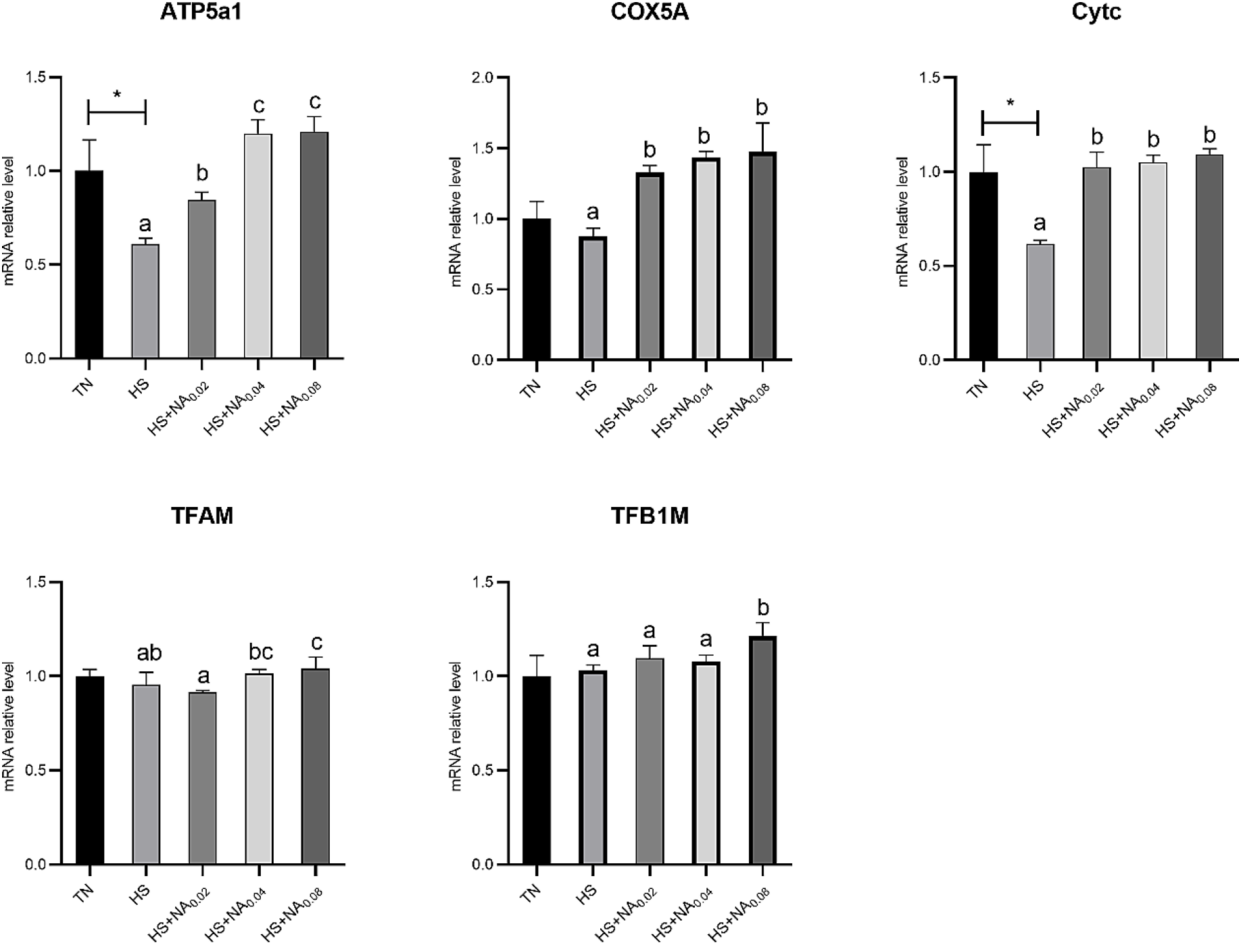
Effect of NA on mitochondrial biogenesis in heat-stressed TBsf. Between HS and HS + NA groups, the difference between different lower letters reached a significant level (*p* < 0.05) and different upper letters reached a very significant level (*p* < 0.01). Not if labeled with the same letter (*p* > 0.05). *p* < 0.05 for the TN vs. HS group is indicated by*.

As shown in Figure 5, heat stress noticeably decreased the mRNA abundance of ATP5A1 and Cytc in TBsf skeletal muscle (*p* < 0.05). Adding NA markedly raised the mRNA expression levels of ATP Synthase F1 Subunit Alpha (ATP5A1), Cytochrome c oxidase subunit Va (COX5A), and Cytc in TBsf muscle (*p* < 0.05). Importantly, adding 800 mg/kg NA markedly enlarged the abundance of mRNAs for TFAM and TFB1M (*p* < 0.05).

## 4 Discussion

COR is a well-known biomarker of heat stress conditions and is essential for assessing the severity of HS and an organism’s response to heat stress (33). Elevated levels of COR indicate a physiological response to stress in animals. Monitoring COR can be an effective way to assess HS levels in poultry. Several reports have demonstrated that heat stress can lead to markedly elevated serum levels of COR in both livestock and poultry. In this experiment, heat stress significantly increased the COR level in the serum of TBsf compared with TN, indicating that the heat stress model for broiler chickens was successfully established. Large amount of research has found that in animals in hot conditions, the body produces large numbers of free radicals and the ability to scavenge free radicals is reduced. Aggregation of large amounts of free radicals can lead to cause protein denaturation and aggregation and trigger lipid peroxidation, which can have serious negative effects on meat quality (34). It was found that intraperitoneal injection of 100 mg/kg NA marked growth the serum levels of SOD, catalase (CAT) and glutathione (GSH) in mice and also attenuated paracetamol-induced oxidative stress (35). Similarly, we found, the addition of niacin significantly improves the antioxidant capacity of heat-stressed broiler muscle while decreasing MDA levels. There are few studies on the antioxidant capacity of NA in broilers, and one study found that niacin increased tissue levels of nicotinamide adenine dinucleotide (NAD) and nicotinamide adenine dinucleotide phosphate (NADPH) (36), an important coenzyme involved in many key life activities and a base for SIRT1. SIRT1 is a highly conserved class of deacetylases that has been shown to attenuate the damage caused by oxidative stress due to iron overload, via nuclear factor erythroid-like factor 2 (Nrf2) (35, 37). Therefore, we suggest that NA enhances the antioxidant capacity of the organism, thereby reducing oxidative stress and improving meat quality.

Tenderness is one of the key indicators of consumer desire to buy. Prolonged exposure to heat increases the diameter of skeletal muscle myofibers, resulting in reduced tenderness (23, 38). Similarly, our research has found that heat stress leads to increased muscle shear. Fortunately, muscle tenderness can be enhanced by dietary addition of NA. While there are many factors that contribute to decreased muscle shear, the composition of muscle fiber types is the most major contributing factor (39). It is well known that the diameter, length and arrangement of myofibers influence meat tenderness. Slow muscle fibers are shorter and finer, thus usually making the meat more tender (40). Therefore, we found that the addition of NA increased the expression of MyHC1 mRNA in heat-stressed broiler muscle. Changes in energy metabolizing enzyme activities may indirectly indicate myofiber transformation (41). It is recognized that type I fibers have higher activities of SDH and MDH, whereas type IIB muscle fibers have higher activities of LDH (42). Heat stress decreases the activity of SDH and MDH (20, 43). This also confirms that dietary NA supplementation promotes myofiber conversion and increases the proportion of type I myofibers. However, more definitive investigations are needed to determine the modulatory mechanisms of NA-induced myofiber type conversion.

Former studies revealed that heat stress resulted in decreased expression of PGC-1α and TFAM mRNA in cardiomyocytes (44), suggesting inhibition of mitochondrial function. In the present study, we found that heat stress resulted in decreased mRNA expression of SIRT1, NRF-1, TFAM, ATP5A1, and Cytc in TBsf skeletal muscle, suggesting that heat stress inhibits mitochondrial biogenesis. A previous study found that niacin increased PGC-1β mRNA levels and enhanced the expression of mitochondria-related genes (45). This is similar to the results of the present experiment, where niacin increased AMPK, SIRT1, NRF-1, TFAM, ATP5A1, Cytc, and COX5A mRNA increased and increases mitochondrial function and number. This may be due to the ability of NA to activate SIRT1, followed by activation of AMPK through deacetylation and indirect stimulation of SIRT1 through increased NAD^+^. SIRT1 also activates PGC-1α, which, once activated (either through phosphorylation or deacetylation), initiates a cascade of NRF-1, and enhances the expression of TFAM. This process affects mtDNA transcription and replication (46), expanding the share of slow muscle fibers and improving meat quality.

## 5 Conclusion

This research provides the first evidence that dietary addition of NA is effective in improving the suppleness, proportion of slow muscle fibers and antioxidant capacity of heat-stressed TBsf leg muscles. Based on the results of the study, dietary addition of 400–800 mg/kg NA is the optimal dose for the treatment of TBsf.

## Data Availability

The original contributions presented in the study are included in the article/supplementary material, further inquiries can be directed to the corresponding author.
